# Impact of the Membrane Structure of the Stationary Phase on Steric Exclusion Chromatography (SXC) of Lentiviral Vectors

**DOI:** 10.3390/membranes13100849

**Published:** 2023-10-23

**Authors:** Jennifer J. Labisch, G. Philip Wiese, Karl Pflanz, John Linkhorst

**Affiliations:** 1 Lab Essentials Applications Development, Sartorius, Otto-Brenner-Str. 20, 37079 Göttingen, Germany; 2 Chemical Process Engineering, Rheinisch-Westfälische Technische Hochschule (RWTH) Aachen University, Forckenbeckstraße 51, 52074 Aachen, Germany

**Keywords:** steric exclusion chromatography, stationary phase, pore size, cellulose membrane

## Abstract

For steric exclusion chromatography (SXC), hydrophilic stationary phases are used to capture the target molecule in the presence of polyethylene glycol. The influence of the structure and pore size of the stationary phase on the process requirements are not yet well understood. To better understand the SXC process, membranes with different pore sizes that served as a stationary phase were compared for the purification of lentiviral vectors (LVs). A design of experiments (DoE) was performed to assess the combined impact of PEG concentration and membrane pore size on the purification performance. A visualization experiment showed that the LVs were captured on the first membrane layer for a pore size up to 2.2 µm, and for a pore size larger than 2.2 µm, LVs were also partly found on the second and third membrane layers. Moreover, we could observe that increasing membrane pore size requires a higher PEG concentration to achieve comparable LV recoveries. Using five membrane layers as a stationary phase was sufficient to achieve good performance, supporting the visualized capture results. In conclusion, we could show that each stationary phase has its optimal PEG buffer compositions for SXC, depending on the membrane structure and pore size.

## 1. Introduction

Lentiviral vectors (LVs) are a powerful tool for delivering therapeutic genes for gene modified cell and gene therapy [1,2]. LVs are currently under investigation in numerous clinical trials [3], with six FDA-approved therapies to date [4]. After LV production using mammalian cells, LVs need to be purified during the downstream process, which involves harvesting and purifying LVs through clarification, chromatography, and ultrafiltration [5]. Producing high virus yields while maintaining quality and purity is a challenge in LV production. Downstream processing is complex due to the lipid envelope of LVs and their large size [6,7]. Due to the increasing demand for cell and gene therapies, cost-effective methods are needed to ensure affordability and accessibility for patients. Steric exclusion chromatography (SXC) is a potential alternative to commonly used chromatography methods such as ion exchange or affinity chromatography, particularly for enveloped viral vectors [8]. As this method does not require any chemical interaction between the target species and the stationary phase, it can be considered a platform technology [9]. This allows for milder elution conditions and preserves viral activity. A detailed description of the SXC purification principle can be found elsewhere [10,11,12]. SXC offers a high potential for viral vector purification and other large sensitive biomolecules [8,13]. The mild process conditions, the high product recovery yields, and the low cost of the method show promising incentives to further develop and understand the method. A considerable number of studies have been performed in recent years, demonstrating applicability for various targets using different stationary phases [8]. The underlying mechanism of SXC is based on PEG-mediated depletion interaction of the target with the stationary phase [12]. For this reason, the impact of the PEG buffer composition (PEG molecular weight and concentration) on the purification performance was intensively investigated in the past [12,14,15,16,17]. Furthermore, different buffer compositions and supplementary salt concentrations were investigated [10,11,15]. Different stationary phases have been used for SXC, mainly membranes and monoliths, and each purification process was optimized based on one stationary phase. Only one study compared different stationary phases but applied the same process conditions for all membranes [14]; thus, the results are difficult to evaluate, as each stationary phase and structure might have its own requirements and optimal conditions, e.g., buffer composition. Later, Eilts et al. hypothesized that smaller membrane pore sizes require lower PEG concentrations in the buffer [15], but the influence of the structure and pore size of the stationary phases is yet not well understood. An in-depth comparative study using different stationary phases or a stationary phase material with varying pore sizes but overall same structure has not yet been performed. In this study, stationary phases of different manufacturers, structures, and pore sizes were compared to better understand the SXC process and to investigate fundamental pore size influences. Understanding the influence of the stationary phase properties on purification process requirements is crucial and offers higher flexibility and predictability of the process.

## 2. Materials and Methods

### 2.1. Lentiviral Vector Production, Harvest, and Clarification

Third-generation lentiviral vectors were produced by transient transfection of suspension HEK293T/17 SF cells (ACS-4500, ATCC) with four plasmids in a UniVessel^®^ 10 L bioreactor operated by a BIOSTAT^®^ B-DCU (Sartorius, Gottingen, Germany). Detailed information on LV production and nucleic acid digestion can be found in [18]. The cell culture broth was clarified using Sartoclear Dynamics^®^ Lab V50 (0.45 µm polyethersulfone membrane version) with 5 g∙L^−1^ diatomaceous earth (Sartorius). The lentiviral vector was aliquoted and stored at −80 °C.

### 2.2. Steric Exclusion Chromatography

#### 2.2.1. Membrane and Housing

**Figure 1:**
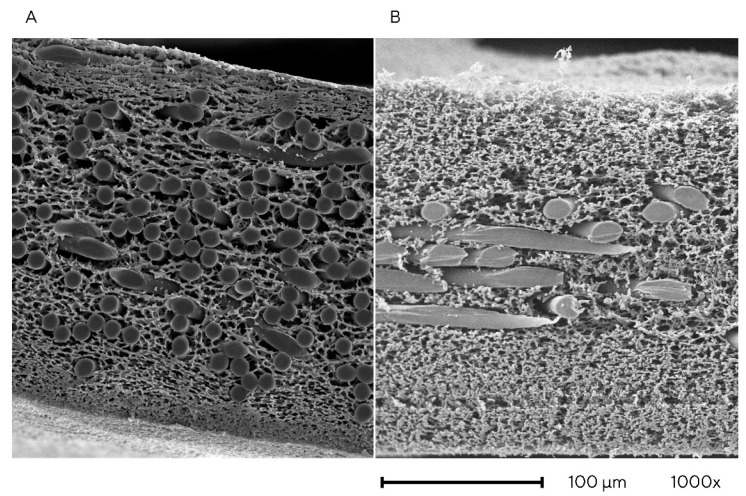
Cryosections of Hydrosart regenerated cellulose (RC) membranes captured with a scanning electron microscope at a 1000× magnification. (**A**) Hydrosart type I. (**B**) Hydrosart type II.

Three different membrane types were used in this study: Hydrosart type I, Hydrosart type II, and Whatman regenerated cellulose membrane. Hydrosart membranes of type I and II (Sartorius) are regenerated cellulose membranes that are additionally reinforced and crosslinked. Figure 1 shows structural differences between the two types of Hydrosart membranes due to different manufacturing processes, resulting in more consistent pore size distribution in type II. Hydrosart membranes with different pore sizes of type II were used (1.4–2.9 µm); type I had a consistent pore size of 2.2 µm.

A fleece structure is incorporated during the production of the membrane to increase overall stability. Hydrosart membrane production, characterization, and the integrity testing of membrane devices have been previously described in Labisch et al. [12]. The membrane lot used in this study had a thickness of 218 µm per layer. The Whatman membrane (Cytiva, Marlborough, MA, USA, 10410014) is a regenerated cellulose (RC) membrane that is not reinforced and has a pore size of 1 µm and a measured thickness of 79 µm. Stacks of five membrane layers were incorporated into an MA15 polypropylene module housing (Sartorius) and overmolded with an injection-molding machine or incorporated into a stainless-steel holder to access membranes easily for LV visualization experiments.

#### 2.2.2. Chromatography Setup and Procedure

The chromatography system ÄKTA™ avant 150 (Cytiva, Marlborough, MA, USA), operated by UNICORN 7.1 software, was used to purify the lentiviral vectors by SXC. All chemicals (Tris, hydrochloric acid (HCl), sodium chloride (NaCl), PEG 4000) were purchased from Carl Roth. Buffers were prepared in ultrapure water of Arium^®^ Pro (Sartorius). These two buffers were prepared: (A) 50 mM Tris-HCl buffer with 150 mM NaCl, pH 7.4 and (B) X% PEG 4000 (depending on the experiment) in 50 mM Tris-HCl, 150 mM NaCl pH 7.4, hereinafter referred to as Tris buffer and PEG buffer. On the day of the experiment, the LV sample was thawed and then kept on ice. The membrane device was first equilibrated with 20 mL of the Tris buffer and the PEG buffer, which were mixed inline in a 1:1 ratio. The PEG buffer with, for example, a concentration of 25% (*w*/*v*) PEG 4000 then reached a final PEG concentration of 12.5%. PEG buffer concentrations varied between the experiments and are indicated in the results section. A total of 25 mL of the LV sample was loaded by inline mixing with the PEG buffer in a 1:1 ratio in the downflow direction (50 mL in total). The wash step was performed as the equilibration step with 15 mL. The LVs were eluted with 25 mL of Tris buffer in the upflow direction. The flow rate was 7 mL∙min^−1^ for every run. The fractions were collected and cooled at 4 °C; after the run, they were aliquoted and stored at −80 °C for analysis.

### 2.3. Analytics

#### 2.3.1. LV Titer, Protein, and DNA Quantification

The infectious LV titer was quantified with the Incucyte^®^ S3 live-cell analysis system (Sartorius). Adherent HEK293T cells (ACC 635, DSMZ) were infected with serially diluted LV samples, and GFP expression was measured by real-time imaging as described in detail in Labisch et al. [19] with the following modifications: no staining was performed, and transgene expression (GFP) was read out 48 h post-infection. Samples were analyzed in duplicates. The LV particle titer was quantified with an enzyme-linked immunosorbent assay using the QuickTiter™ Lentivirus titer kit (Cell Biolabs, San Diego, CA, USA). The total protein concentration was determined with the Pierce™ Coomassie Bradford protein assay kit (Thermo Fisher Scientific, Waltham, MA, USA). The total dsDNA amount was quantified with the Quant-iT™ Pico-Green™ dsDNA assay (Thermo Fisher Scientific). All assays were performed according to the manufacturer’s instructions.

#### 2.3.2. Lentiviral Vector Visualization

Staining was performed to visualize the location of the LV on the membrane. One LV batch containing 1.0 × 10^7^ TU∙mL^−1^ was incubated for 1 h at 4 °C with a mouse monoclonal antibody to VSV-G (F-6), labeled with Alexa Fluor^®^ 546 (Santa Cruz Biotechnology, Dallas, TX, USA), in a dilution of 1:2000 (final concentration 0.1 µg∙mL^−1^). Five membrane layers were incorporated into a MA15 housing in a stainless-steel holder. The screws were tightened to 3 Nm. The SXC run was performed as described above and stopped before elution. The membranes were separated and visualized with a UVP ChemStudio (Analytik Jena, Jena, Germany) by applying the green light source (550 nm), the ethidium bromide filter, and an exposure time of 60 s. An untreated membrane layer that was not incorporated into the membrane holder device was used as a negative control.

### 2.4. Statistical Analysis

The statistical significance of between-group differences was evaluated using an unpaired Student’s *t*-test (two-tailed) with OriginPro^®^ Version 2021 (OriginLab). Where applicable, experiments were evaluated with MODDE Pro 13 (Sartorius).

## 3. Results and Discussion

### 3.1. Impact of Membrane Pore Size on the Separation Mechanism

In a recent publication, the capture of the LVs on the membrane was investigated, and it was found that the LVs were mainly captured on the first membrane layer [18]. We were further interested in discovering if the location of the captured LVs was influenced by the membrane structure and pore size, in order to gain further insights into the separation mechanism. Therefore, two different regenerated cellulose membranes from two different suppliers (Cytiva and Sartorius) were compared. Additionally, two different membrane structures of the stabilized regenerated Hydrosart cellulose membrane (Sartorius) with different pore sizes were compared. LVs were labelled with a fluorescent antibody and loaded on the membrane that was incorporated into an MA15 housing and placed in a stainless-steel holder.

**Figure 2:**
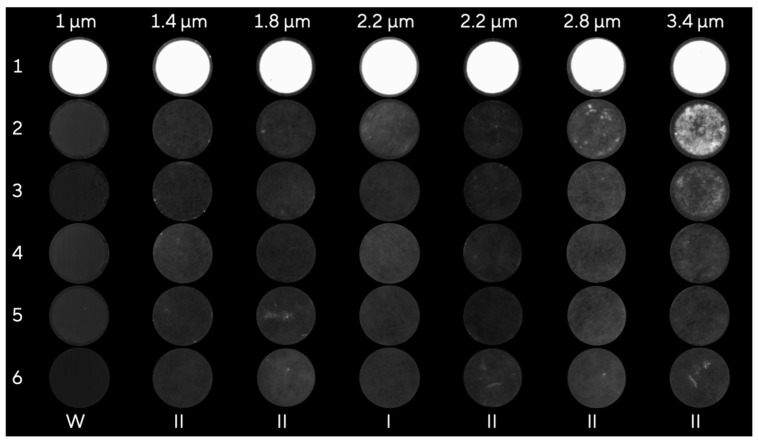
Comparison of the membranes of a stacked chromatography module. Row numbers indicate layers 1–5 ordered from top layer to bottom layer. The pre-wetted membrane layer serves as a negative control (row 6). Whatman (W) and Hydrosart type I and II (I, II) membranes were compared with different pore sizes indicated at the top.

Potentially unbound antibodies are removed in the flow-through due to the large size difference of IgG (15.5 × 8.5 × 4.0 nm [20] and LVs (80–120 nm diameter [5]. The pronounced size difference allows for the selective retention of the larger LV particles, as discussed in more detail elsewhere [8,12]. Therefore, it can be assumed that unbound antibodies are not retained and efficiently removed in the flow through fraction. The same loading conditions were applied for all membranes: a final PEG 4000 concentration of 12.5% and a flow rate of 7 mL·min^−1^. A volume of 50 mL was loaded, corresponding to a 25 mL LV solution mixed in a 1:1 ratio with a PEG buffer. The loading volume was defined based on previous experiments on membrane capacity [18]. The SXC runs were stopped before the elution step, and the five membrane layers were removed from the MA15 chromatography device and compared against a pre-wetted control membrane without LVs. The results of the experiment are summarized in Figure 2.

LV particles were present on the first membrane layer for all pore sizes up to 2.2 µm. Furthermore, we observed that the Hydrosart I membrane with a pore size of 2.2 µm had some LVs on the second layer, whereas the second membrane layer of the Hydrosart II showed no signal. This can be explained by the more homogenous membrane structure and narrower pore size distribution. From a pore size of 2.8 µm, individual bright dots can be seen on the second membrane layer. The largest pore sizes examined show the highest fluorescence on the second layer and slight punctual fluorescence on the third layer.

As previously described [18], it can be concluded that 10–20 membrane layers, as often described in the literature [14,21,22,23], are not required for efficient purification and that the separation takes place in the first membrane layer. The influence of the membrane height on the purification performance is further evaluated in Section 3.3. Moreover, a membrane layer number of three might be sufficient for small pore sizes of around 1–2 µm, whereas membranes with larger pores like 3–5 µm would require five membrane layers to avoid breakthrough. It was possible to show that the membrane structure and pore size have an influence on the location where the target was captured. Based on these findings, elution in an upflow direction is beneficial, as the eluted target does not have to pass through the entire membrane depth during elution. Understanding the capture location aids in understanding the fact that five membrane layers are sufficient; thus, material can be saved. A more detailed discussion of how this affects the scaling of the process can be found in [18].

### 3.2. Membrane Pore Size and PEG Buffer Concentration

**Figure 3:**
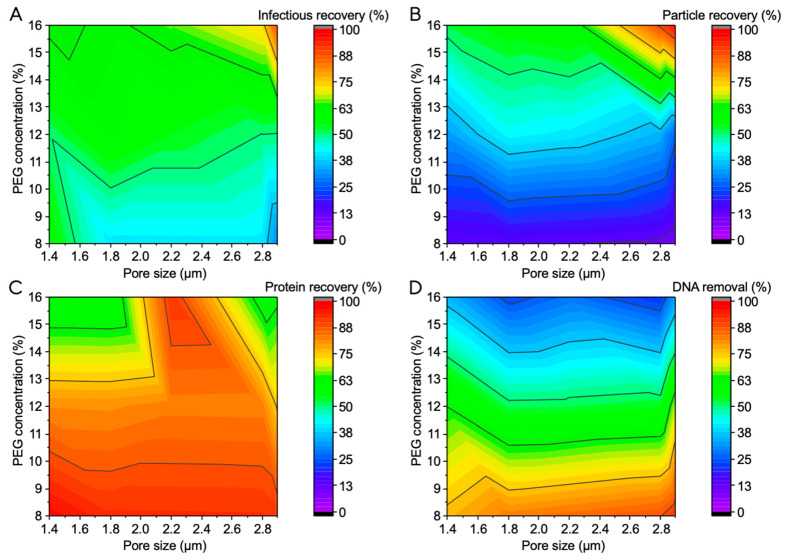
Contour plots of the factors membrane pore size and PEG buffer concentration for the responses (**A**) infectious recovery, (**B**) particle recovery, (**C**) protein removal, and (**D**) DNA removal.

With the aim being to analyze the influence of different membrane pore sizes in combination with different PEG buffer concentrations, a design of experiments (DoE) approach was performed, which consisted of 16 runs, including three center points. Pore sizes from 1.4 µm to 2.9 µm and PEG 4000 concentrations from 8% to 16% were tested. Five layers of Hydrosart II membranes were used and incorporated into an MA15 device. The results are presented in contour plots in which the PEG concentration was plotted against the utilized membrane pore size (Figure 3). The measured percentages of the respective recovery or removal were areal interpolated and matched to a color scale from 0–100%.

The highest LV recovery of 88% was achieved with the largest pore size tested of 2.9 µm and the highest PEG concentration of 16%. When analyzing the contour plots, it becomes apparent that with small pore size, low PEG concentrations are sufficient to achieve a recovery of approximately 60%. As the pore size increases, the PEG concentration required to achieve the same recovery also increases. Therefore, it can be concluded that the PEG concentration in the buffer or the molecular weight of the PEG has to be adjusted depending on the stationary phase properties. Generally speaking, with increasing membrane pore size, a higher PEG concentration is required to achieve an efficient depletion interaction of the viral particles with the membrane. A similar trend was observed for particle recovery. This can be explained by the fact that the polymer concentration affects the steric exclusion of colloidal particles. A single particle in a solution experiences a uniform osmotic pressure. But when the particle comes close to the stationary phase, PEG molecules cannot penetrate the gap, resulting in a negative osmotic pressure, thus resulting in weak attraction between the LV particles and the stationary phase. The increase in the polymer concentration leads to a rise in the osmotic pressure, thus leading to an increase in the attraction of LV particles and the stationary phase, making their association with the stationary phase more probable [10,11,12,14,24]. When one visually imagines how LV particles pass through a membrane, it becomes clear that the probability of a particle encountering/colliding with the solid membrane structure via random movement decreases with a larger membrane pore size. By increasing the PEG concentration, this effect can be compensated, as the osmotic pressure is increased, which leads to a stronger attraction force of the LV particles and the membrane. The impact of the membrane structure apart from the membrane pore size remains unclear and requires further investigation.

DNA removal showed an opposing trend compared with LV recovery. With increasing PEG concentration, the DNA removal decreased. A similar trend was observed for the protein removal but achieved generally higher values compared with the DNA removal. The DNA removal at process conditions at which high LV recoveries are achieved were lower than the values of other SXC processes described in the literature, which achieved over 80% DNA removal [14,22,23,25]. A reason for this could be the size of the DNA molecules present. Due to insufficient DNA digestion during upstream, longer molecules (larger hydrodynamic radius) would not be properly depleted, as steric exclusion would affect them. Moreover, the use of suspension cell lines in this study results in an increased impurity load, complicating the downstream process, compared with the aforementioned studies relying on adherent cell lines. Thus, further optimization of DNA digestion is required.

### 3.3. Performance Comparison Using Different Membrane Layer Numbers

**Figure 4:**
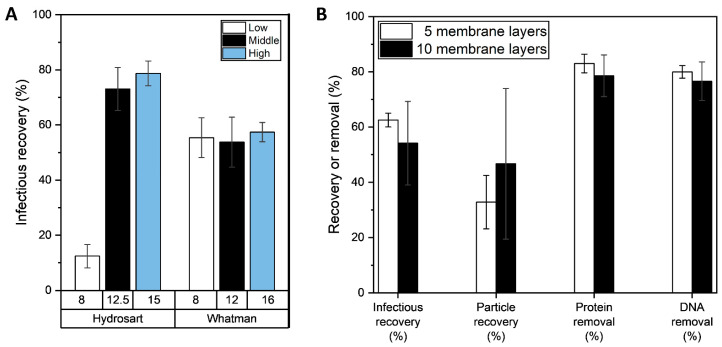
(**A**) Direct comparison of different stationary phases (Hydrosart I membranes and Whatman membranes) on infectious recovery for different PEG concentrations. The color coding indicates the relative PEG concentration; the exact PEG concentration is indicated below the *x*-axis. (**B**) Comparison of SXC performance with 5 or 10 layers of Whatman membranes. Analytical parameters were statistically compared between the two tested membrane stacks using a paired *t*-test of two different samples (*p* ≥ 0.05) with no significant differences found. N = 3, mean ± standard deviation.

Five layers of a Hydrosart membrane (type I, 2.2 µm pore size) and the Whatman RC membrane (1 µm pore size), which are the two membranes predominantly used for SXC [8], were used to purify clarified LVs, using three different PEG concentrations. The highest infectious LV recovery of 73–79% was achieved with the Hydrosart membrane in combination with a 12.5% or 15% PEG buffer (Figure 4). By contrast, the LV recovery was significantly lower, using a final PEG concentration of 8%. The Whatman RC membrane showed a similar LV recovery for all PEG buffers tested, which was around 55%. A possible explanation for this could be the different pore sizes of the membranes (2.2 µm vs. 1 µm) as well as their different structures, which affects separation efficiency. As previously discussed in Section 3.2, a larger membrane pore size requires a higher PEG concentration for efficient LV purification. We expect that this effect can be observed with the Whatman RC membrane when using lower PEG buffer concentrations than the ones examined in this study. For the Whatman RC membrane (pore size 1 µm), the final PEG buffer concentration of 8% was already sufficient, and a further increase in the PEG concentration did not increase the infectious LV recovery, as observed for the Hydrosart membrane. No intensive process optimization with the Whatman RC membrane was performed; thus, further adjustment of the buffer composition or the evaluation of other flow rates might be useful for this membrane type. In general, it becomes clear that each stationary phase, depending on its structure and chemistry, has its optimal process conditions, and the SXC purification process must be optimized depending on the stationary phase used. Once the stationary phase properties are changed, the process conditions may have to be adapted.

The height of the membrane and its associated surface volume is a crucial factor in the process design of many chromatographic or filtration processes. In previous publications, the Whatman membrane was often used with 10–20 layers [8]. To analyze whether this might be crucial for this type of membrane, the height of two membranes was measured to assess whether a certain membrane layer number but bed height might be necessary. The Hydrosart membrane has a height of 218 ± 4 µm in a dry state and 229 ± 1 µm in a pre-wetted state (increase in thickness by 5%) due to their reinforcement structure, whereas the Whatman membrane has a lower height of 79 ± 1 µm in a dry state and 91 ± 7 µm in a pre-wetted state (increase in thickness by 15%) and is more prone to swelling, as it is a non-stabilized cellulose membrane. Hence, to achieve the same bed height, more membrane layers are required for the Whatman membrane. MA15 modules incorporated in a stainless-steel holder were prepared with 5 or 10 layers of Whatman RC membranes, and an SXC was performed using 12% PEG as capture buffer. SXC purification of LVs with the Whatman RC membrane achieved an infectious recovery of 54–63% and a particle recovery of 32–46%. The impurity removal was over 77%. No significant differences were obtained between 5 or 10 membrane layers, and a higher standard deviation was observed when using 10 membrane layers. This result is in accordance with the staining experiment in Section 3.1, which showed the usage of the first membrane layer with the Whatman RC membrane as for the Hydrosart type I membrane. Labisch et al. also observed no significant differences in the LV recovery for SXC with 5 and 10 layers of a stabilized cellulose membrane (Hydrosart I) under identical process conditions [12]. Therefore, it becomes clear that the membrane height (for the membrane height range tested here) has no decisive influence on the process, and five membrane layers are sufficient for application and are recommended to save material. Although the first two layers are the ones mainly essential for capture, we used at least five layers to achieve uniform fluid flow across the membrane.

## 4. Conclusions

Steric exclusion chromatography (SXC) utilizes hydrophilic stationary phases and polyethylene glycol-containing buffer to capture target molecules. However, the influence of stationary phase structure and pore size on process requirements remained unclear. To address this, we compared membranes with different pore sizes for lentiviral vector (LV) purification. Visualization experiments revealed LV capture only on the first membrane layer, with pore sizes up to 2.2 µm, and partly on the second and third layers for larger pores. Furthermore, we confirmed that five membrane layers as a stationary phase are sufficient, as the target is predominantly captured on the first 1–2 membrane layers. Using five membrane layers enabled a uniform fluid flow across the membrane. Increasing pore size required higher PEG concentrations for comparable LV recoveries. But it must also be considered that higher PEG concentrations were associated with lower impurity removal, especially DNA. In conclusion, each stationary phase requires optimal PEG buffer compositions for SXC that are dependent on membrane structure and pore size.

## Data Availability

Not applicable.
